# Mobile Laser Scanning Systems for Measuring the Clearance Gauge of Railways: State of Play, Testing and Outlook

**DOI:** 10.3390/s16050683

**Published:** 2016-05-12

**Authors:** Sławomir Mikrut, Piotr Kohut, Krystian Pyka, Regina Tokarczyk, Tomasz Barszcz, Tadeusz Uhl

**Affiliations:** 1 Department of Geoinformation, Photogrammetry and Environmental Remote Sensing, AGH University of Science and Technology, 30-059 Cracow, Poland; krisfoto@agh.edu.pl (K.P.); tokarcz@agh.edu.pl (R.T.); 2 Department of Mechatronics and Robotics, AGH University of Science and Technology, 30-059 Cracow, Poland; pko@agh.edu.pl (P.K.); tbarszcz@agh.edu.pl (T.B.); tuhl@agh.edu.pl (T.U.)

**Keywords:** mobile laser scanning, measurement of clearance gauge, photogrammetry, database

## Abstract

The paper contains a survey of mobile scanning systems for measuring the railway clearance gauge. The research was completed as part of the project carried out for the PKP (PKP Polish Railway Lines S.A., Warsaw, Poland) in 2011–2013. The authors conducted experiments, including a search for the latest solutions relating to mobile measurement systems that meet the basic requirement. At the very least, these solutions needed to be accurate and have the ability for quick retrieval of data. In the paper, specifications and the characteristics of the component devices of the scanning systems are described. Based on experiments, the authors did some examination of the selected mobile systems to be applied for measuring the clearance gauge. The Riegl (VMX-250) and Z+F (Zoller + Fröhlich) Solution were tested. Additional test measurements were carried out within a 30-kilometer section of the Warsaw-Kraków route. These measurements were designed so as to provide various elements of the railway infrastructure, the track geometry and the installed geodetic control network. This ultimately made it possible to reduce the time for the preparation of geodetic reference measurements for the testing of the accuracy of the selected systems. Reference measurements included the use of the polar method to select profiles perpendicular to the axis of the track. In addition, the coordinates selected were well defined as measuring points of the objects of the infrastructure of the clearance gauge. All of the tested systems meet the accuracy requirements initially established (within the range of 2 cm as required by the PKP). The tested systems have shown their advantages and disadvantages.

## 1. Introduction

The measurement of the structural gauge of railway lines results from the necessity to build a system for their codification in Poland by 2030. By that time, all railway lines should be covered by such a system. This problem has existed since the 1990s, when the first studies of the construction for such systems was being developed for the transport of cargo with an excessive loading gauge and combined transport gauge.

As part of a research project carried out for PKP (PKP Polskie Linie Kolejowe S.A., Warsaw, Poland) in 2011–2013 under the title, “Development of Innovative Methodology and IT Management System for the Codification of Railway Lines”, AGH University of Science and Technology conducted experiments involving the search for the latest solutions relating to mobile measurement systems that meet the basic requirement (accurate and the ability for quick retrieval of data, measured at speeds of around 100 km/h). 

The scientific goal of the research was to propose a methodology for measuring the structure gauge of railway lines, based on the literature of the existing solutions and testing of selected systems. The following research tasks have been completed in this project:

- obtain photogrammetric data in the aspect of the spatial modelling of the clearance gauge of railway buildings and structures,
- develop a methodology for building a spatial vector model of the railway infrastructure clearance gauge,
- automatically texture elements of the space that describe the clearance gauge of railways,
- develop a methodology for updating the clearance gauge of objects at railway lines,
- develop a spatial structure for the database of the railway infrastructure clearance gauge,
- develop a methodology for the determination of the kinematic clearance gauge of cargos,
- develop a method for the interactive assignment of codes to railway lines,
- develop the assumptions and structure of an IT system for managing the process of assigning codes to railway lines.

In order to achieve this goal, the following tasks had to be completed:

- a review of the literature,
- selection of the appropriate testing systems,
- selection of the track sections for measurement tests, preparation of test fields and performance analysis of the measurements under the test experiments.

The experiments conducted were meant to be based on systems involving laser scanners and photogrammetric cameras. In turn, they would provide an answer to the question about the advantages and disadvantages of individual solutions; in addition, whether an accuracy of 2 cm in the cross-section perpendicular to track axis, as assumed by PKP, would be met. 

Following the provisions of the Master Plan for Railway Transport in Poland until 2030, all railway lines of the target network, on which cargo trains shall be travelling, should be subjected to codification. Those lines, which are designed exclusively for passenger traffic (*i.e.*, high-velocity lines or metropolitan area lines) should not be subjected to codification. 

The basic assumption for the application of the system of encoding railway line sections is a complete and up-to-date clearance gauge database concerning railway structures. It also includes codes that will be assigned to railway line sections, the issue of permits for the transport of special cargos and the special conditions for these transports will be established.

It is not possible to maintain a complete and up-to-date clearance gauge database concerning railway structures without an efficient, mobile system for clearance gauge measurements.

The following article is a summary and brief description of the attempts made, along with a discussion of the results.

## 2. Review of the Existing Mobile Systems, Both the Ones Dedicated to Clearance Gauge Measurements and Other Universal Mobile Scanning Systems

The first mobile scanning systems emerged in the 1990s. The scanning devices currently available on the market are mounted on vehicles travelling by land, sea or air. Such systems feature a high integration of various types of sensors and the automation of data acquisition. 

Every mobile scanning system consists of two components: data-receiving sensors and navigation equipment. The most commonly-used sensors include digital cameras and laser or radar scanners. For the basis of measurements, one obtains the spatial location of points, relative to the scanning platform, through the calibration of measuring systems in relation to geo-referenced systems and/or in an external frame of reference. For scanners, this is based on direct measurement, and when using a vision system, this is completed by analyzing and measuring the characteristics of the images. 

A navigation system of a vehicle consists of a GPS receiver and an INS inertial navigation module, which continuously monitors the position and orientation of the vehicle. The GPS receiver is the primary device that measures the position of the vehicle, and it uses a network of satellites of the Global Positioning System. A limitation of the applicability of GPS navigation involves the potential blocking of the satellite signal and the low frequency of determining the orientation of the vehicle. The relative position and orientation of the sensors comprising the measuring system is obtained by integrating the signals coming from the INS module. The readings from the INS must be adjusted by input data from an external source. The necessary adjustments are provided by the GPS. In addition, the system may include other sensors, such as odometry, inclinometers and barometers.

Today, standard mobile systems include laser scanners and digital cameras that, when combined, create color point clouds, which allow the possibility to automatically identify objects in the cloud. Classical photogrammetry is also used and automation is possible with the latest matching algorithms (matching of features), detection and identification of objects in digital images. The algorithms used here include LSM (Least Square Method) and the latest ones: BRIEF (Binary Robust Independent Elementary Features), SIFT, SURF, FAST. Thanks to the improvements made to devices for geo-referencing, the absolute accuracy of the system is already high and amounts to about 1–2 cm. Scanning systems are used for 3D mapping of cities, creating 3D models of architectural objects, positioning and testing of deformations of road infrastructure, automatic recognition of road signs [1], measurements of rail infrastructure, including the measurement of the clearance gauge, *etc*.

Currently, the operating systems are mainly based on three groups of methods:

(1) A method based on the measurement by means of a laser rangefinder and a laser scanner (LiDAR),
(2) Photogrammetry, using a stream of images,
(3) A method involving light profiles projected by a light laser and recorded by a fast digital camera.

The most advanced systems combine a number of the above-mentioned measurement methods.

Examples of mobile systems based on laser scanning include: Riegl MLS VMX-450 [2], Mitsubishi MMS-X220 [3], ROAMER [4], Zoller + Fröhlich Profiler [5], LIMEZ II/III [6,7], L-KOPIA/LKO B1 Clearance Laser System [8], Street Mapper [9], Topcon (IP-S2 HD) [10,11] and ROAMER [12]. There are also other MLS scanner geometries that take into account movement and 3D mapping [13,14].

The systems based on the photogrammetric method include: Limez II [7], Trimble [15], GPSVision [16], GeoAutomation [17], GeoVISAT [18], OmniVision [19], Earthmine—Mars Collection System [20], Leador2000 RMMS [21] and GeoVan [22].

An example of systems based on the principle of light profiles registered by vision devices are: Balfour Beatty Rail [23] and MSVS (multi-camera and structured-light vision system) [24].

Apart from the commercial systems for mobile 3D measurement, there are a number of interesting systems constructed by research teams from scientific institutions [25,26,27,28]. Some of the mobile systems are dedicated especially to the measurement of the clearance gauge (Balfour Beatty Rail, LIMEZ II and III, Zoller + Fröhlich Profiler) or the railway infrastructure (Railmapper) [29]. L-Kopia is a two-way system, whereas Riegl and 3D Mapping Solution have such a function built into the application. An example of solving the problem of measuring the clearance gauge in tunnels was shown in [24]. The authors presented an example of a system for clearance gauge measuring that was based on seven CCD cameras and structured light projectors (MSVS) and, additionally, on two cameras and two projectors. Calibrations of the various systems are briefly outlined below.

## 3. Specifications and Characteristics of the Component Devices of Scanning Systems

### 3.1. Characteristics of Scanners 

The scanners can be broken down into one of the following criteria:

- Measurement principle (triangulation, polar measurement),
- Distance measuring method (phase and pulse measurement),
- The maximum measuring range (microscanners, short-, medium- and long-range scanners),
- Field of view (profile, widescreen, camera, hybrid view),
- Accuracy (low >10 mm, average: 1–10 mm, high <1 mm).

Systems based on laser scanning have been selected for these tests. During the test measurements, we tried to examine various types of scanners in order to check their properties (possible advantages and disadvantages for mobile measurements) and the impact these properties have on the accuracy and speed of measurement. 

The selected scanners have an accuracy of a few millimeters at a distance of several meters (e.g., scanner Faro FOCUS 3D at a distance of 10 m has an error of 2 mm).

The manufacturer of the tested Riegl VMX 250 system claims that the system accuracy is 10 mm when measured at a distance of 50 m with 5-mm precision (measurement repeatability). 

As a rule, mobile systems use one scanner for measuring the profile perpendicular to the direction of travel or two (or more) scanners angled so as to cover an area in the form of a grid.

### 3.2. Characteristics of IMU Systems

IMUs (Inertial Measurement Units) are designed to determine the angular orientation of the measuring device in 3D space (in our case, the entire scanning system). The orientation can also be used to control rotating objects. IMU makes it possible to measure the instantaneous linear accelerations and angular velocities of said objects. An IMU consists of accelerometers and gyroscopes.

The IMU operating frequency is within the range of 100 Hz–3600 Hz, depending on the technology of the accelerometers and gyroscopes (laser, optical, MEMS technology) used.

An IMU system with software is called an INS (Inertial Navigation System).

IMUs also have software that makes it possible to adjust the measurement system to the dynamics of the measurement object, to visualize the measurement data in real time and to register the data. 

The orientation of the device is defined by specifying the Euler angles, referred to as the “roll” (rotation around the axis in line with the direction of travel), the “yaw” (rotation around the vertical axis) and the “pitch” (perpendicular to the other two). The measurements of IMU “navigation” angles are made in relation to the locally-assumed system centered at the center axis of the sensor system, the local vertical line (*Z*-axis) and the direction of the travel axis of the measuring system (*Y*-axis). This system is time-varying in line with a change in the position of the system to form an inertial frame of reference (referred to as the “body”).

The estimation of the Euler angles is based on the idea of sensory fusion, in which one algorithm integrates the measurement data from various sensors. The angles can be determined from the gyroscopes by the matrix integrating of angular velocities and from the accelerometers and magnetometers, using algebraic relations. The IMU devices are used for specifying the angle elements of the measuring system, in this case a mobile scanning system. IMU-type devices feature the determination of angles with various accuracies. Such systems are usually coupled with GNSS-type receivers. 

The accuracy of inertial navigation is affected in the first place by the accuracy of the measurement of angular velocities. The breakdown of inertial units by the angle achieved via the accuracy of the inertial system is parameterized by the gyro drift values. 

Inertial measurement units used in mobile measurement systems belong to two accuracy groups:

- Navigation units: drift amounting to 0.001–0.100 degrees/h,
- Tactical units: drift amounting to 0.1–100 degrees/h.
- GNSS/IMU: POS 420 V4 LW, a positioning system made by Applanix, supported by DMI, is the leading solution on the mobile systems market.
- The tested systems, e.g., RIEGL (VMX 250), identified the angles with the following accuracy:
- Roll and pitch angles: 0.005 degrees,
- Heading angle: 0.015 degrees.

### 3.3. Characteristics of the Vision Systems

Vision sensors in mobile systems dedicated to clearance gauge measurements are usually used for three tasks:

1. Coloring of a point cloud acquired by means of a laser scanner,
2. Supervision and registration of the measurement process,
3. Photogrammetric measurement of gauge points.

Currently, research is underway on the use of images from such systems for automatic detection and identification of objects in railway infrastructure. 

Vision systems are characterized by parameters that describe the geometrical model of the camera, their radiometric features, the characteristics of an image carrier and the interface between the camera and image acquisition cards. 

The camera’s geometric model is presented by means of the internal parameters represented by a matrix called a calibration matrix, which describes the transformation between the coordinate system of an image and the coordinate system of a sensor. It is determined in the process of camera calibration and described by the imaging distance, the location of the principal point of an image in the imaging coordinate system, the pixel size in the *x* and *y* axes and its skew ratio, lens distortion model coefficients, along with the radial and tangential distortion. 

The geometric parameters of the camera are needed both when using the images for coloring a point cloud and for photogrammetric measurement, with the latter application requiring more accuracy of their designation. 

The vision sensors used for measuring the gauges work under boundary lighting conditions and show objects both in deep shadows and in full sun. The automated selection of exposure parameters under dynamic conditions does not prove efficient. Therefore, the sensor’s radiometric features, such as sensitivity, radiometric resolution (tonal range), spectral resolution and spectral range can be of decisive importance when choosing a device.

The radiometric features of sensors are affected by the lens-matrix array. Basic radiometric features of a sensor, such as sensitivity, radiometric resolution and spectral resolutions, are listed in the sensor’s specifications. The sensitivity of the camera is, as a rule, higher when operating in the monochrome mode than when operating in the RGB mode; the standard tonal range is eight bits per channel, yet often, higher ranges can be found (10, 12 and 14 bits per channel).

Such features, as sharpness, contrast and resolution, are determined by the modulation transfer function or the spatial frequency response, which informs about the optical system’s response depending on the spatial frequency of the signal. 

The ratio of the maximum amount of light that the sensor can capture to the minimum amount of light is the dynamic range of the sensor. The maximum level of the signal depends on the capacity of the detector, while the minimum level is an effect of noise. Cameras of high dynamic ranges can record at the same time both very strongly-illuminated objects and very deep shadows. Tonal range is the number of signal levels within the dynamic range. Tonal response is the relation between the signal and the amount of light, shown in the form of a graph. Gamma is a slope of the tonal response curve, and that slope is the contrast index. 

Another feature to consider is the accuracy of color rendering, which is measured and calculated on the basis of differences between reference colors and the colors rendered by the sensor. 

Noise is an effect of accidental changes in image pixels’ brightness, manifesting itself in deviations from the average level for the same reference brightness as imaged by the sensor. The measure of noise is the average standard deviation, determined for various brightness levels. The noise level of sensors is listed in the detailed camera specifications, but one can examine it based on tests involving a standard color or gray level target. 

Digital cameras generally use two types of photosensitive matrices: CCD and CMOS. Sensor matrices differ, first of all as regards sensor sizes, sensitivity, type (color or monochrome), readout rate, special features (*i.e.*, windowing, binning).

CCD sensors make it possible to render high-quality images of very low noise levels, while CMOS sensors are more susceptible to noise and are marked by a lower sensitivity (they have low pixel filling ratios). Unlike CCD sensors, CMOS sensors consume less power (over 100-times less than CCD sensors). One can manufacture CMOS sensors on nearly any standard production line, resulting in them being significantly less expensive as compared to CCD sensors.

CCD sensors are dedicated for cameras that are designed to produce images of high quality and resolution, with an excellent luminous sensitivity. Due to lower noise levels, CCD sensors are beneficial to obtain low-contrast images. The main advantage of CMOS sensors is their high versatility. They are capable of windowing in order to sense a lower amount of data at a considerably higher acquisition speed increase. They are also main elements of quick digital cameras, which acquire images with a frequency of even one million frames per second, e.g., Phantom v12. 

Digital cameras furnished with various CCD photosensitive matrices can operate using line-by-line, interlaced or progressive scanning modes, whereas CMOS matrices complete sensing sequences in “rolling shutter” or “global shutter” modes. For measuring objects that are in motion, CMOS cameras with global shutter exposure are recommended. Image acquisition frequency ranges from several to even a million frames per second (Phantom v12). Presently, resolution ranges in camera matrices exceed 25 Mpix (e.g., Bobokat IGV-B6620 of a resolution of 29 Mpix). Interfaces used at digital cameras include USB, FireWire, CameraLink, GigE and CoaXPress, while the digital standards most commonly applied in vision systems are USB 2.0, IEEE 1394a and 1394b (FireWire), CameraLink and Gigabit Ethernet (GigE).

### 3.4. The Conception of the Database

The project framework also included the development of the database conception, knowing that data obtained from laser scanning would require much space (1 km of railway route equals over 1.5 GB of data).

It was the task of AGH to work out an IT solution that matched existing systems already in place at PLK. An IT solution has been developed, including the database, an application to browse the database and to assign codes, as well as a number of auxiliary programs designed for importing measurement data in the database. The database constitutes the key element of that solution. Following the review of databases that are available on the market, a decision was made to utilize Oracle.

In addition, before proceeding with the project works, the authors made the following assumptions:

- The database must be capable of importing data directly from measurements, so it must record both point clouds and images. At the same time, data from point clouds is to be analyzed first, while image data is to be used in the event that an area holds some doubts (e.g., areas where the information provided by the point cloud alone is not 100% sufficient to make a decision).
- The database must cooperate with the POS (Leading Network Description) and SILK (Railways Lines Information System) databases. In both of these databases (POS and SILK), the basic way of addressing objects at railway lines is that of chainage. The requirement of the project was the connection of our database with POS and SILK, to exchange the data.
- Due to compatibility with the SILK database, data would be processed under the 1992 national geodetic coordinate system, yet the database must be capable of converting data to other coordinate systems.
- When developing the database, the following input data were utilized:
- GPS/INS data, including the vehicle trajectory coordinates; *ca.* 200 samples per second.
- Laser scanning data (a point cloud), about 25 million points (~1.5 GB) per kilometer of railway line.
- Images (in JPG format) taken by four cameras; about 350 images (~0.3 GB) per kilometer of railway line.

**Figure 1:**
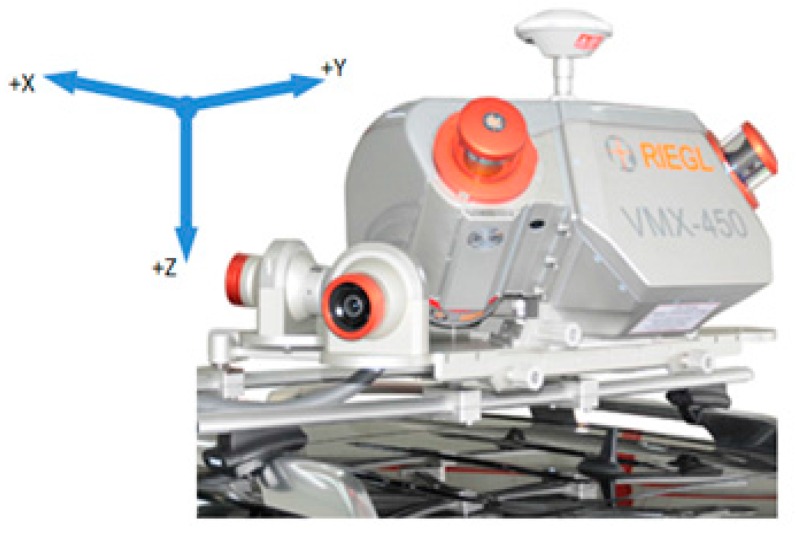
The coordinate system of the RIEGL VMX-250. Axis *X*, vehicle travel direction; *Y*, vertical on top of rails and pointing up; *Z*, perpendicular to the *XY* plan in the nadir direction.

During the process of designing the database, several procedures were developed. These procedures were aimed predominantly at reading in the data, preliminary reducing of their volume, then simplifying and re-arranging the data layout, so that other processes can be carried out more efficiently. By doing so, this ensures quicker database responses connected with the visualization of data and the assigning of codes. The most important items of that stage included the following:

- Calibration of chainage: Chainage is the basic mode of addressing/indexing railway line objects. Mapping the geographic coordinates to a Linear Referencing System (LRS), which is used in the SILK, can be made with the use of the Oracle Spatial software with dynamic segmentation functions (3D to 1D transformation). The present LRS model for railway lines in the SILK database was made with a cartographic accuracy of 1:25,000 maps. Therefore, a decision was made only to calibrate data based on a dozen or so points obtained from the SILK database.
- Determination of track geometry: Based on GPS data, the examined railway line sections were divided into elementary sections of a length of 1 m each. Assuming that the turning radius is neglectable within such a section, the location of both rails was identified in the point cloud (together with rail height, which made it possible to make allowance for super elevation in further considerations). In this way, the local system of coordinates was determined. Furthermore:
  ○ the system’s center was precisely positioned vertically just over/on the rails in the *Y* axis, between the rails in the *Z* direction and halfway of the section (1 m) along the traveling direction (*X*) (right-handed system),
  ○ axis *Y* was positioned vertically on top of rails and pointing up (making allowance for super elevation),
  ○ axis *X* was perpendicular to *Y* and overlapping the vehicle travel direction,
  ○ axis *Z* was perpendicular to the *XY* plane (see Figure 1).
- Reduction of data: In order to reduce the amount of 3D data stored in the database, a decision was made to limit the point cloud to strips along the travelling path, being 9.5 m wide (four to the left, four to the right side and 1.5 m as the distance between rails).
- Considerations regarding the implementation of 2D cross-sections: For each identified 1-m section of the line, a projection of the point cloud upon the *YZ* plane was made. As each of such sections has its own system of reference, as defined above, and each of those systems is describing space from the viewpoint of a moving vehicle, it is possible to combine such cross-sections. The project participants decided to store in the database cross-sections from 10-m, 100-m and 1-km sections, as well as total cross-sections connected with encoding principles described in the International Union of Railways UIC 502-2 chart and dividing a railway line into segments (*i.e.*, those between railway stations or turnouts).

#### 3.4.1. Conception of Railway Clearance Gauge Determination

The conception of railway clearance gauge determination as designed in the IT solution is largely based on the 2D contours method. 

As 3D cross-sections based on point clouds are considerably large in size, compression is necessary. Storing such cross-sections may prove to be impractical. On the other hand, the accuracy of such cross-sections (of a single point) is unnecessary from the point of view of the clearance gauge (and its codes). Therefore, as part of the project, an algorithm was developed to create 2D cross-sections on data from point clouds (through flattening of 3D point cloud data).

A 2D cross-section derived from a point cloud in the referencing system related to the track axis is created through dividing the whole 2D area into an exemplary 50 × 50 mm grid. If there is any point within the selected grid cell, the whole cell is considered to be occupied.

The algorithm makes such cross-sections at every 10, 100 and 1000 m within any section of the track. Next, those cross-sections get simplified by assuming a grid of 20 × 50 mm. The choice of which interval one should use depends on the kind/type of track (a straight line or turn) and the density of objects around the track (for a straight section with only fields, we use a 1-km cross-section; for dense building data and platforms, we use a 1-m cross-section).

That operation can be performed in an automatic manner. The application makes it also possible to combine cross-sections, creating a single cross-section within a section of several kilometers.

The task of the program operator is to review cross-sections and, if any conflict is identified (clearance gauge limits exceeded), to request the database to provide a more detailed cross-section or an image showing the suspicious area.

**Figure 2:**
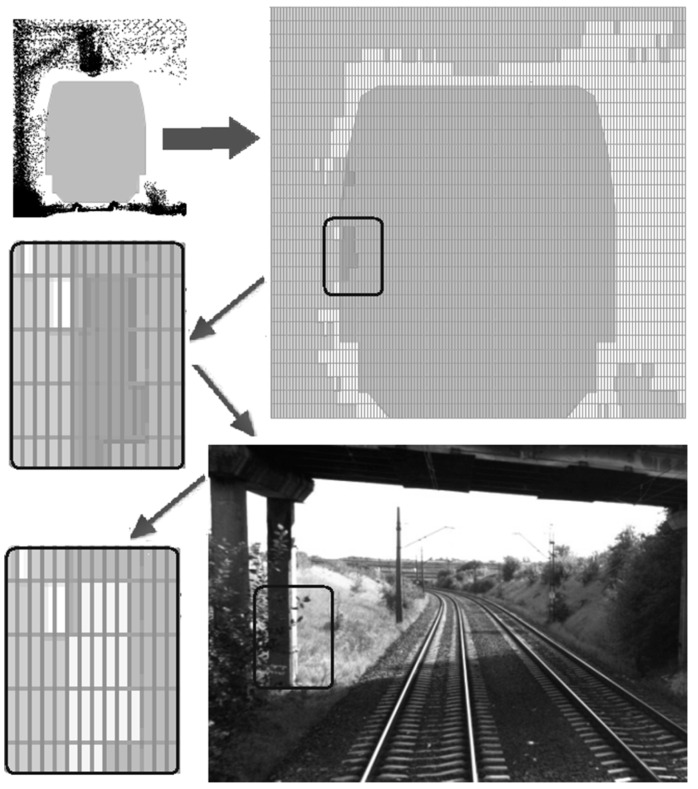
Managing cross-sections in the database. The small outlined region (top right) is 20 × 50 mm. This region contains data where the cross clearance data and scanner data overlap, triggering white rectangles that are shown by the system (lower left picture). The operator should check this region to determine the source of the overlap. The operator can retrieve image data (lower right) to check/verify; in this case, the questionable area is the branch.

Based on the data, the operator may invalidate any element of the grid. The diagram of that conception is shown in Figure 2.

Figure 2 shows an exemplary cross-section, made with a resolution of 20 × 50 mm. It can be seen that clearance gauge limits of the structure have been exceeded (dark grey color at the light grey background). In such a case, it is necessary to verify the reasons. Here, thanks to visualization, these are only some plants that one can see in the images. 

In such a situation, the grid points may be unchecked as “invalid” (light grey).

#### 3.4.2. Assigning of Codes 

The project made use of the contour method originating from the UIC 502-2 chart. That method is marked by a more careful selection of base points.

**Figure 3:**
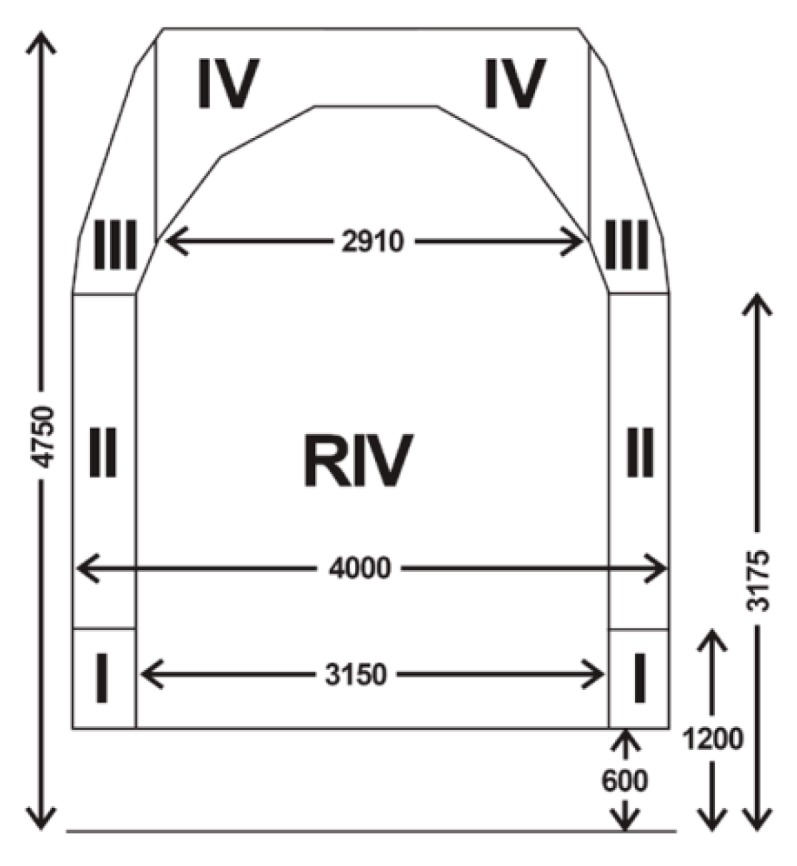
Determination of the section numbers in the clearance gauge. Dimensions in mm. RIV is code of international trade.

In the database solution, there were procedures developed to implement that method. Furthermore, a method of the interactive assignment of codes to the railway line was developed. To maintain compatibility with the UIC chart, the possibility of dividing the railway line into sections was introduced. To facilitate the search, sections were assigned numbers (Figure 3).

Furthermore, the possibility of checking if a cargo can be allowed for shipment was added, this was provided through the implementation of the critical points method. The points describe in an approximated way the shape of the cargo, however maintaining the rules to guarantee that the cross-section of the cargo would not be smaller than the actual shape of the cargo. The method that has been implemented makes it possible to calculate the cargo parameters, considering several calculation variants.

In the case of cargo, a procedure of automatic single code assigning was developed. Thanks to that procedure, the criterion of accepting the cargo for transport comes down to comparing the cargo code, railway line code and what is being transported: e.g., rail line Code 3-3544-465 allows for any cargo Codes 0-0444-425, but does not allow for 0-0466-425, as below in the Table 1.

Through the extensions, it is possible to examine the encoding effectiveness through verification of the results that are based only on comparing codes to graphic visualizations of the route cross-sections and of the cargo available in the extended viewer.

## 4. Examination of the Selected Mobile Systems in the Application for Measuring the Gauge 

The system was adapted to allow the installation of the measurement systems to be examined; specifically, a platform wagon was adapted. The wagon can be pulled by a locomotive with a maximum speed of 100 km/h. Such an arrangement makes it possible to use a standard rail platform, to mount system components in variant mutual configurations and to scan almost full 360° profiles. 

### 4.1. Characteristics of the Tested Measuring Systems 

Two systems have been chosen as representative for test measurements:

- A system based on a phase scanner profiling in two directions perpendicular to the direction of travel (System 1),
- A system based on two pulse scanners profiling in two directions diagonal to the axis of the track and forward, with integrated cameras (System 2).

The goal of using System 1 was to verify the measurement technology by means of a device dedicated to the railway. It uses a Z+F PROFILER 9000 profiling scanner coupled with an odometer. The system operates in data acquisition mode in planes perpendicular to the direction of the train travel within 360° and makes measurements in the track axis arrangement.

**Figure 4:**
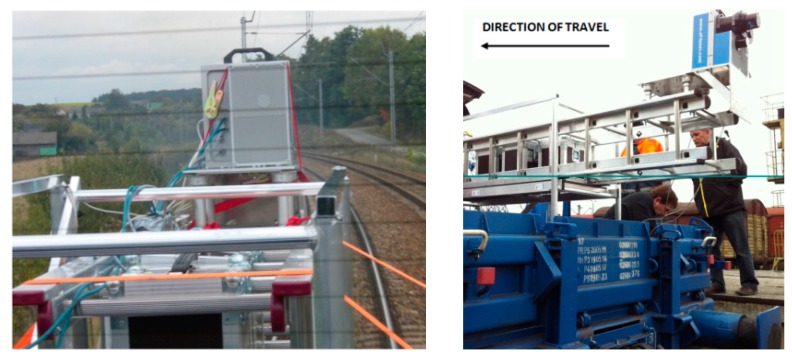
System 1: Z+F Profiler 9000 scanner on a railroad carriage. In the picture, the installation of the Z+F scanner and construction on the platform are shown ((**left**) rear view; (**right**) side view).

**Figure 5:**
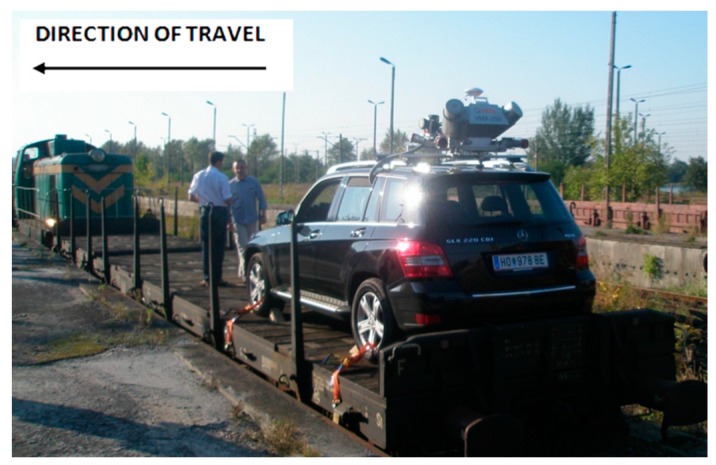
System 2 based on a solution by RIEGL VMX-250 mounted on the platform.

The scanner Z+F Profiler is a good choice for stop-go inspections of specific locations. It was used in tandem with an R088ND3 odometer made by Wenglor. In order to carry out the test measurement, it was placed on a platform (RES-type wagon), on a previously-prepared structure (Figure 4). The Z+F PROFILER 9000 is a phase scanner that makes it possible to record over one million points per second with a maximum recording speed of 200 profiles per second. Thanks to such parameters, it is possible to obtain very small distances between the cross-sections, even at high speeds of the travelling platform. However, sometimes, we have flat discs perpendicular to the track. In that case, the element is not measured. System 2 (Figure 5) was based on a solution by RIEGL VMX-250, designed mainly for mobile measurements of roads and highways. 

This is a unit consisting of two RIEGL VQ-250 scanners scanning diagonally to the direction of travel, including a GPS, which are installed under an aerodynamic cover. It also consists of four digital cameras used for coloring a cloud of points whose range of vision can be defined for each camera individually, depending on the requirements. The entire system was located on the roof of a car, which was placed on a wagon platform. This system enables the geo-referencing of a point cloud in a reference frame adopted for GPS measurements. 

The idea of the system was presented during the International ISPRS Congress in Melbourne in 2012 [30]. 

### 4.2. Field Experiments 

Test measurements were carried out within a thirty (30)-km section of the Warsaw-Kraków route. Areas were then selected so as to provide various elements of railway infrastructure, a varied geometry of the trail (straight line, turn) and the installed geodetic control network. This makes it possible to reduce the time of the preparation of geodetic reference measurements for the testing of the accuracy of the selected systems. The reference measurements were made on selected profiles perpendicular to the axis of the track.

The reference points were well defined (easy to measure and identification) as the objects of the infrastructure of the clearance gauge.

Besides this, GPS technology was used to measure the coordinates of checkpoints signaled by means of surveying poles. The measurements were carried out by means of a Laser-Tec track gauge made by the company Graw, which provided millimeter accuracy for positioning of the points (relative to the axis of the track). Laser-Tec is designed to measure equipment and facilities of a size greater than 10 mm in diameter, including semaphores, tunnels, platforms and the space between the width and height of the contact wire. A similar measurement accuracy was provided by the measurement of checkpoints by means of a total station. A GPS reference measurement of checkpoints signaled by poles provided a coordinate accuracy of approximately 1 cm. 

The measurement by means of the Riegl System (System 2) took place at a platform speed of about 80 km/h, with the exception of the route sections with a lower speed limit. The measurement by means of the Z+F Profiler (System 1) took place twice: at a velocity similar to that of the first system and at a velocity of about 40 km/h. The measurements were carried out alternately on both tracks of the route, which resulted from travel conditions. 

The results of the measurements made by means of the tested systems produce point clouds in various arrangements; System 1 makes it possible to obtain gauge profiles in planes perpendicular to the axis of the track, whereas the cloud of points from System 2 is expressed in the 2000 system through satellite positioning. This point cloud can be used to transform to the railway gauge arrangement, provided that the axis of the track is defined.

**Figure 6:**
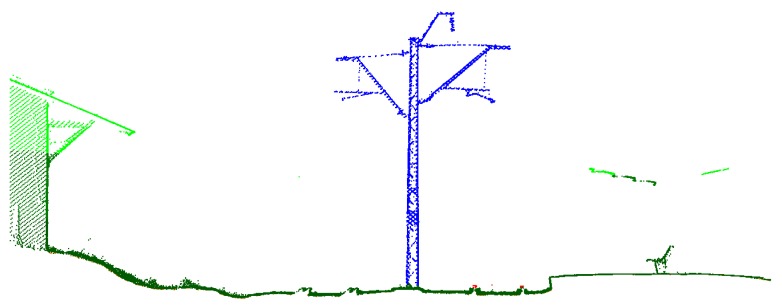
Cross-section through the railway station at Słomniki, obtained with System 2. We see the point clouds after classification (dark green, lower part; bright green, average height; red, rails; blue, traction pole).

**Figure 7:**
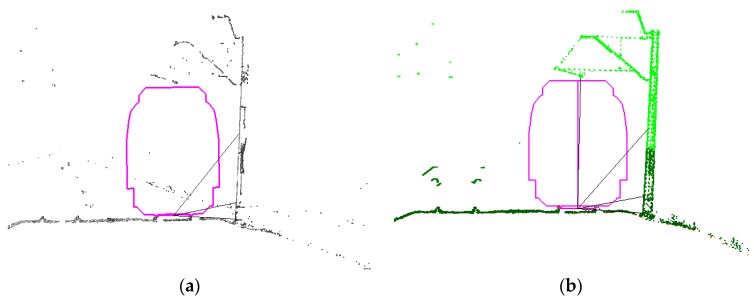
Clearance with measured railway infrastructure elements obtained from rail gauge measurements: (**a**) in the background, the cloud of points from the Z+F Profiler 9000 system; (**b**) in the background, the cloud of points originating from the RIEGL VMX-250 system.

Therefore, the verification of the accuracy of System 1 involved a comparison of cross-sections with reference clearance gauges in the same planes, whereas the verification of the accuracy of System 2 was made with regard to the designation of gauge profiles and external accuracy tested at checkpoints. Exemplary cross-sections are shown below (Figure 6 and Figure 7).

**Figure 8:**
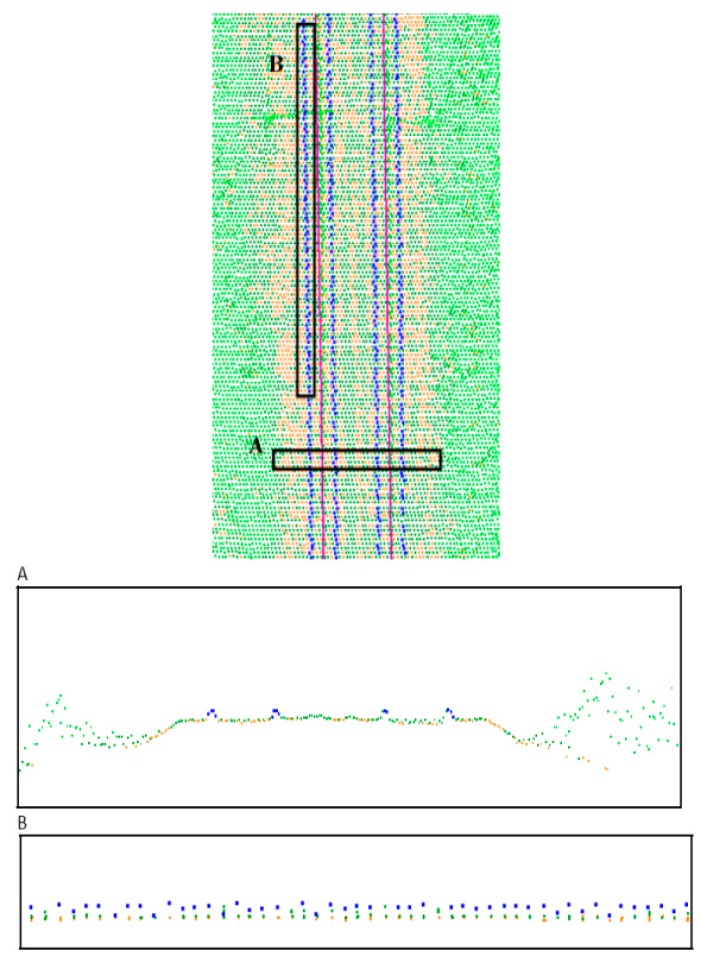
Cross-sections with automated railheads done by Terrasolid. (**A**) Perpendicular to the track; (**B**) Along the track; extracted automatically.

The track axis for the measurements of both systems was determined on the basis of laser measurements obtained after automatic filtration of cross-sections of railheads. In order to process the point cloud from both systems, we used the following software: for System 1, it was a Z+F Laser Control, and for System 2, it was RiPROCESS, whereas Terrasolid was used for automatic extraction of the track axis for the measurements of both systems (Figure 8). 

A comparison of measurements in cross-sections by means of System 1 with reference measurement provided the mean differences in section planes (Table 2). 

Respectively for System 2 (Table 3): 

High standard deviations on profiles are caused by a difference in the method used for determining the axis of the track in the tested systems and in the reference system, which affects the accuracy of determining points in the profiles. The track axis in System 1 and System 2 was determined from a profile obtained from a point cloud to railheads. 

The absolute accuracy of points (details of the infrastructure objects) as measured by means of System 2 at checkpoints resulted in the following results (Table 4).

The reference points were measured by means of a total station instrument.

At the points measured by means of GPS (Table 5): 

### 4.3. The Database Application

The effectiveness of the final encoding of railway lines translates into the verification of the whole system operation correctness. The project authors were supposed to provide also an application, which would effectively be utilizing measurement data and calculating clearance gauge values.

Therefore, an application was developed for an interactive presentation of the collected data, with an option of checking if a cargo can be allowed for shipment and travelling along the selected railway line section. In addition to assigning codes to railway lines, as discussed above, the following should be mentioned as being the most important functionalities of the application:

- generation of animations

**Figure 9:**
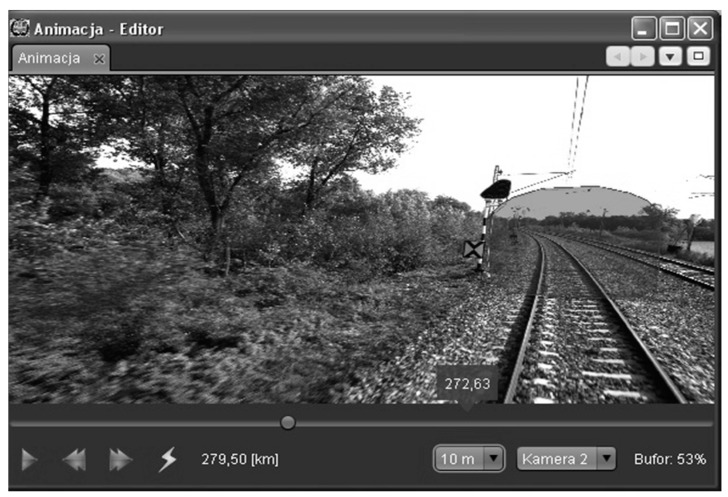
The animation window in the implemented program.

Figure 9 presents an example of an animation. The animation is being generated on a current basis of images downloaded on-line from the database (including the necessary buffering). One can pick up images from one of the available cameras and additionally place/superimpose on the film any freely-selected contour. This can be a pre-defined contour template (*i.e.*, an outline of the structure’s limiting edge or an outline of the international railway clearance gauge) or a contour generated in other parts of the application (*i.e.*, the shape of the cargo). The navigation bar is indexed with the route chainage. One can move into any place of the route, stop the animation and change the camera. After the animation has been stopped, it is possible to open another view, containing a 2D cross-section or a view of a 3D point cloud.

- point cloud visualization

**Figure 10:**
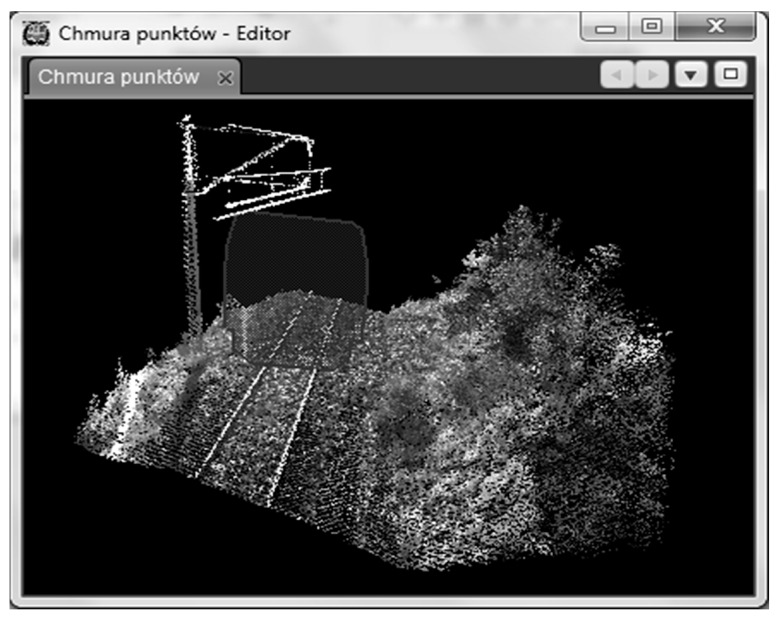
A 3D view presenting a point cloud.

Figure 10 presents a typical visualization of the point cloud of a selected route fragment. It is possible to add any contour template to that view that has been defined in other parts of the application. Using the mouse, one can move that cross-section to any place along the track. The goal of placing a contour template on that view is to assist in identifying those clearance gauge elements that interfere (collide) with that contour. Moreover, by using the mouse, one can perform such operations as changing the view’s perspective, rotating the view, enlarging it, *etc.* The view also makes it possible to measure a distance between any two points or to read the coordinates of the selected point. Measurements can be made both in the track system and in the geodetic system. The view is synchronized with other views, such as those in the image viewer.

- image viewer

**Figure 11:**
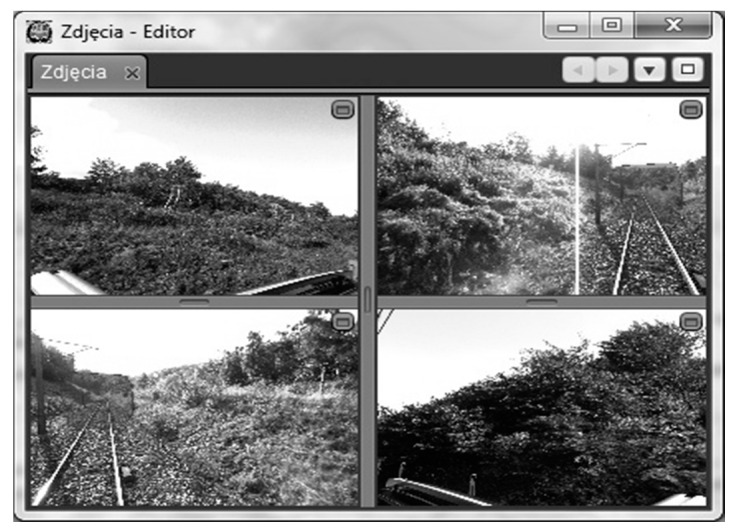
Image viewer. We see the pictures from cameras installed on the RIEGL system and set up in four directions.

Figure 11 presents an image viewer that has been integrated in the program. The view is synchronized with a 3D view. The contour template that has been marked on images is moving and is the same contour of the 3D view. In this way, using images, one can better identify those clearance gauge elements that collide with the marked contour. Images are provided with chainage segment identifiers, and the time stamps of image acquisition are available as an image attribute. It is possible to monitor images from all four cameras or from a single one only.

- visualization of 2D cross-sections

**Figure 12:**
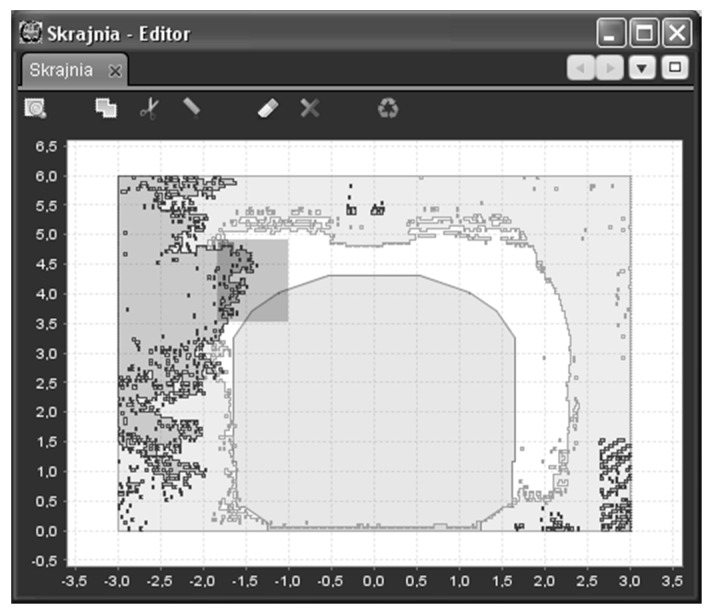
A 2D cross-section: clearance gauge and the point cloud around. Coordinates of the axes are in meters.

Figure 12 presents a 2D cross-section view as implemented in the program. The view shows a cross-section made through a longer section of the line, with an isolated 10-m cross-section, represented by the darker color (center). By using that view, one can compare/superimpose a cross-section (Figure 9) made through a selected section of the route with the selected contour template (*i.e.*, the shape of a cargo), make clearance gauge measurements in the track axis system, look for interfering or colliding elements or uncheck non-significant elements (*i.e.*, plants).

- entering the cargo

**Figure 13:**
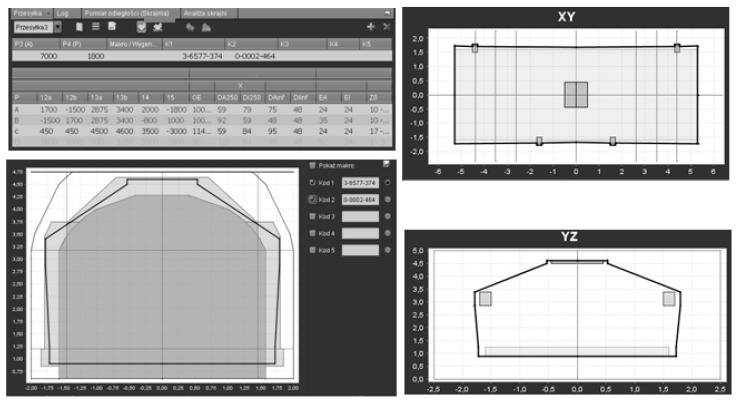
Cargo geometry and clearance editor/profiler. Coordinates of the axes are in meters. Figure presents the critical points method which makes it possible to enter the shape of cargo (**lower and upper left**). On the basis of points that have been entered, cross-sections in the *XY* (**upper right**) and *YZ* (**lower right**) planes are generated.

Figure 13 presents the critical points method, which has been integrated in the program and which makes it possible to enter the cargo. On the basis of points that have been entered, cross-sections in the XY and YZ planes are generated. This makes it possible to choose various options for calculating the necessary allowances as described in the UIC 502-2 chart and in the Ir-10 Instructions. Based on those options, one can generate the shape of the cargo that may later be subjected to the process of cargo code assigning. More information about the database conception and the project itself can be found in [31].

## 5. Conclusions

The point of these tests was to answer the question of whether the choice of laser scanning as the methodology for measuring the structure gauge of railway lines is correct and whether the tested measuring systems meet the accuracy requirements. On the basis of a broad literature review, we selected systems appropriate for testing and conducted the test experiments: we chose the test sections where test measurements were prepared and conducted. 

It can be concluded on the basis of the results shown in Section 4 that the tested systems meet the accuracy requirements established at the beginning (accuracy within 2 cm). The adopted research methodology proved to be correct. Each tested system has its own advantages and disadvantages.

System 1 obviously has an essential advantage, which is the ability to perform the measurement in the plane in which the clearance gauge is measured. This is possible because the scanner scans in a profile, and therefore, it is perpendicular to the direction of travel. Unfortunately, it has its limitations because the distance between the profiles depends on the velocity and varies from several to a dozen or so centimeters; therefore, there is a probable risk of omissions in the measurements of such infrastructure elements that are essential and whose dimension along the axis of the track is small and, hence, might not be measured, e.g., elements such as discs, which are set perpendicularly to the train travel direction, may have several millimeters in width.

This problem can be reduced by means of System 2, where scanners are arranged relative to each other at an angle and cover the scanned area in the form of a grid. However, the disadvantage of this solution is the necessity to move between the 2000 system and the track system for measuring the clearance gauge.

We also verified the accuracy of measurements in the conducted experiments by means of scanning methods. 

The authors of the study have aligned the observations in both systems and compared the coordinates. As can be seen in the presented results, the deviations of both systems were within the accepted limits of 2 cm.

As a result of the project, the database of railway infrastructure spatial data was developed. The database parameters make it possible to import and compile results of measurements originally obtained from various sources. It also allows for them to be updated, to process them according to algorithms that have been devised for the needs of railway clearance gauge measurements, as well as to present spatial visualization of the results, with an option for an interactive, precise measuring of situational details. On the basis of that database and data entered into the system, codes can be assigned to railway line sections, consents to transport of extraordinary cargos can be issued and the conditions of the transport of such cargos can be established.

The authors also plan to publish in future the results of the other tasks mentioned in the Introduction, such as:

- experiments concerning a point cloud filtration, integration of scanning and photogrammetry and an automatic extraction of edges and special curves,
- research on the current state of play of the knowledge on the effective operation of algorithms of the automatic texturing of railway clearance gauge elements,
- designing a model of a ground-based scanner platform, which was then used in a process of the obtaining and updating of the data necessary for developing a spatial model of railway infrastructure,
- research on the prototype for the database of the railway infrastructure clearance gauge, followed by mathematical analyses necessary in the process of railway codification,
- research for the use of photogrammetric measurements for modelling patterns of the kinematic clearance gauge of cargos,
- developing a methodology for an interactive encoding of a railway line at different variants of cargo transport.

## Figures and Tables

**Table 1:** An example of a rail line code (3-3544-465) compared to cargo codes (0-0444-425 and 0-0466-425).

**Rail Line Code**	**3**	**3**	**5**	**4**	**4**	**465**
Cargo Code	0	0	4	4	4	425
Results	OK	OK	OK	OK	OK	Ok
**Rail Line Code**	**3**	**3**	**5**	**4**	**4**	**465**
Cargo Code	0	0	4	6	6	425
Results	OK	OK	OK	Not	Not	Not

**Table 2:** A comparison of the measurements in cross-sections by means of System 1 with the reference measurement. dx is the difference between the tested and the reference measurement in the plane of the clearance gauge in the horizontal direction; dy is the difference between the tested and the reference measurement in the plane of the clearance gauge in the vertical direction.

	Standard Deviation		Arithmetic Mean	
	dx (m)	dy (m)	dx (m)	dy (m)
Track 1	0.021	0.024	0.004	−0.002
Track 2	0.008	0.016	0.002	−0.005

**Table 3:** A comparison of the measurements in cross-sections by means of System 2 with the reference measurement. dx and dy are as in Table 2.

	Standard Deviation		Arithmetic Mean	
	dx (m)	dy (m)	dx (m)	dy (m)
Track 1	0.017	0.026	0.004	−0.005
Track 2	0.014	0.023	−0.002	−0.001

**Table 4:** The absolute accuracy of points as measured by means of System 2 at checkpoints. dxy are differences in the horizontal plane; dz are differences in altitude.

	Standard Deviation		Arithmetic Mean	
	dxy (m)	dz (m)	dxy (m)	dz (m)
Track 1	0.006	0.005	0.019	0.010
Track 2	0.005	0.002	0.025	0.008

**Table 5:** The points measured by means of GPS.

	dxy (m)	dz (m)
Standard Deviation	0.021	0.006
Arithmetic Mean	0.019	0.025
